# Protective measures are associated with the reduction of transmission of COVID-19 in Bangladesh: A nationwide cross-sectional study

**DOI:** 10.1371/journal.pone.0260287

**Published:** 2021-11-22

**Authors:** Nadim Sharif, Khalid J. Alzahrani, Shamsun Nahar Ahmed, Rubayet Rayhan Opu, Nayan Ahmed, Aeken Talukder, Raju Nunia, Mysha Samiha Chowdhury, Israt Jahan Nodi, Tama Saha, Ming Zhang, Shuvra Kanti Dey

**Affiliations:** 1 Department of Microbiology, Jahangirnagar University, Savar, Dhaka, Bangladesh; 2 Department of Clinical Laboratories Sciences, College of Applied Medical Sciences, Taif University, Taif, Saudi Arabia; 3 Department of Epidemiology and Biostatistics, College of Public Health, University of Georgia, Athens, Georgia, United States of America; Chinese Academy of Medical Sciences and Peking Union Medical College, CHINA

## Abstract

Coronavirus disease 2019 (COVID-19) pandemic has become a major public health issue globally. Preventive health measures against COVID-19 can reduce the health burden significantly by containing the transmission. A few research have been undertaken on the effectiveness of preventive strategies such as mask use, hand washing, and keeping social distance in preventing COVID-19 transmission. The main aim of this study was to determine the association of the preventive measures with the reduction of transmission of COVID-19 among people. Data was collected during January 06, 2021 to May 10, 2021 from 1690 participants in Bangladesh. A validated questionnaire was used to collect both the online and offline data. Chi-square test and logistic regression analyses were performed to determine the association among the variables. The prevalence of COVID-19 was 11.5% (195 of 1690) among the population. Age, gender, occupation and monthly income of the participants were significantly associated with the likelihood of following the preventive measures. The risk of infection and death reduced significantly among the participants following preventive measures (*p* = .001). The odds of incidence was lower among the participants using masks properly (OR: 0.02, 95% CI: 0.01–0.43), maintaining social distances (OR: 0.04, 95% CI: 0.01–0.33), avoiding crowded places (OR: 0.07, 95% CI: 0.02–0.19) and hand shaking (OR: 0.17, 95% CI: 0.09–0.41). This study suggests that preventive health measures are significantly associated with the reduction of the risk of infection of COVID-19. Findings from this study will help the policymakers to take appropriate steps to curb the health burden of COVID-19.

## Introduction

Severe acute respiratory syndrome coronavirus 2 (SARS-CoV-2) is the causative agent of the ongoing pandemic, coronavirus disease 2019 (COVID-19) [1, 2]. Individuals with infection of SARS-CoV-2 in the respiratory tract were first identified at Wuhan, China in late December, 2020. The virus has been transmitted rapidly throughout the world [1, 2]. On March 11, 2020, the World Health Organization announced COVID-19 as a pandemic. The COVID-19 pandemic has become one of the major health burdens with continuous increase of cases and fatalities globally [1, 2]. As of June 13, 2021, about 170 million cases and 3.8 million fatalities have been documented [3, 4]. Besides, active and passive immunizations, different protective health measures play significant roles in preventing the transmission of infectious diseases like COVID-19 [5–7]. The World Health Organization (WHO) and Centers for Disease Control and Prevention, USA (CDC) have recommended the common people to follow several preventive measures namely, wearing face masks, maintaining social distance in public places, avoiding gatherings, cleaning hands with soaps, avoiding frequent touch to mouth and nose in communities with patients of COVID-19 [6–8].

Though vaccination against COVID-19 has been initiated in many countries, accomplishment of mass vaccination within short period remains difficult in most of the countries. Studies suggested that protective measures even after vaccination are required to reduce the transmission effectively [5–9]. Most of the countries have adopted special policies regarding the COVID-19 pandemic to reduce the health burden. Accordingly, in Bangladesh, the policy makers have introduced guidelines to reduce the spread of the virus. ‘No mask no service’ policy has been implemented in private and public offices, business centers, educational institutes, universities and shopping malls. Previous studies have found that preventive health measures such as wearing masks, washing hands and maintaining social distance are significantly associated with the reduction of transmission of cases and fatalities [9–13].

Studies have found that the route of transmission for COVID-19 may be nasal passageway. The origin of transmission of COVID-19 remain unclear till now [14–18]. Previous studies suggest that safety measures such as wearing masks can reduce the spread significantly for both the droplet and airborne transmission of infectious viruses including influenza and SARS-CoV [7–12]. The significance of protective measures including use of masks needs to be studied in details to understand the contribution in reducing the spread of COVID-19 in the communities [17–20]. However, protective clothing including hand gloves, personal protective equipment (PPE), face masks and safety goggles have reduced death and infection among the frontline health care providers and doctors [21]. The association of the preventive measures with the reduction of the transmission of COVID-19 needs to be analyzed among the general people.

Second wave of COVID-19 has struck Bangladesh more severely than before. The community transmission of COVID-19 has been reported throughout the country. The detection rate of positive cases has increased to 20% during the second wave from 3–4% of the first wave [3, 4]. In a densely populated country with a large number of daily workers, it is hard to impose and maintain strict lockdown. However, policymakers had imposed lockdown from the first week of April, 2021 and became stricter to monitor and ensure that people following the preventive health measures. This study was conducted to investigate the association of the preventive measures with the reduction of transmission of COVID-19 in Bangladesh.

## Materials and methods

### Study area and period

A web based cross-sectional study was conducted from January 06, 2020 to May 10, 2021 during the second wave in Bangladesh. Data were collected from 1690 individuals. Data were collected from eight divisional cities covering 54 districts in Bangladesh.

### Data collection tools, procedures and variables

Data were collected by interviews over phone calls and by using digital questionnaire. Data couldn’t be collected by face-to-face interviews due to the COVID-19 pandemic situation. The questionnaire was developed in the Bengali language (the mother tongue of the local people). Data collection was conducted by using the same questionnaire all over the country by simple random sampling approach. Sample size was determined by using previously published works [22]. Objectives of the study were demonstrated to the participants and informed consent were taken from them before enrolling in the study.

The investigation was carried out by using a validated questionnaire [23]. Data was obtained by sending out the invitations to the participants via email, Facebook, WhatsApp, YouTube, and Instagram with a link of the questionnaire. For the purpose of data analyses, only complete responses were used. The questionnaire was scored by following the rules in the ‘NHS Patient Survey Program: Scoring of Questionnaires.’ Responses from the participants were also included for the scoring of the questionnaire.

There were five sections in the questionnaire, namely, section A, B, C, D and E. Five questions about the participants’ socio-demographic characteristics were included in Section A. Section B comprised of nine questions about using masks, hand sanitizers and maintaining social distance and following protective measures. Section C, D and E included 23 questions about the RT-PCR tests, case reports, disease prognosis and fatality report to assess the association of the protective measures in containing the transmission of COVID-19 among the participants.

The independent variables in this study included socio-demographic characteristics such as age, sex, profession, monthly income, educational background, family member, residence areas, access to health care of the participants, presence of comorbidity etc. The dependent variables included the practices of preventive health measures and outcome of the pandemic like new cases, hospitalization and fatality. Categorical variables (yes vs no) were also included for the quantitative analyses.

### Ethical approval

Appropriate ethical clearance was taken from the Biosafety, Biosecurity & Ethical Committee at Jahangirnagar University. The approval number of this study is BBEC, JU/M 2021/COVID-19/(8)1.

### Statistical analysis

Descriptive statistical analysis, namely percentages, frequencies, mean, and standard deviation (SD) were calculated for the socio-demographic characteristics of the participants, COVID-19 related health information, preventive measures taken by the participants and government. Data in this study were checked for normal distribution by Kolmogorov–Smirnov test and presented in histogram and pie chart. For conducting the inferential analysis, the non-parametric tests were computed for data that were not normally distributed. The Chi-square test was used for determining the association between demographic factors with the tendency of following the preventive measures. The level of association and the relationship between the categorical independent variables, the preventive health measures and dependent variables such as outcome of COVID-19 infection and fatality was determined by a Chi-square test. A *p* value <0.05 was considered statistically significant. Further, logistic regression analysis was also performed for determining the level of significant association between the independent variables and health outcomes of the participants. Statistical analysis was performed by using the International Business Machines (IBM) Statistical Package for the Social Sciences (SPSS) version 26.0 (Chicago, IL, USA) and Microsoft Excel 2019.

## Results

### Socio-demographic characterization

**Fig 1:**
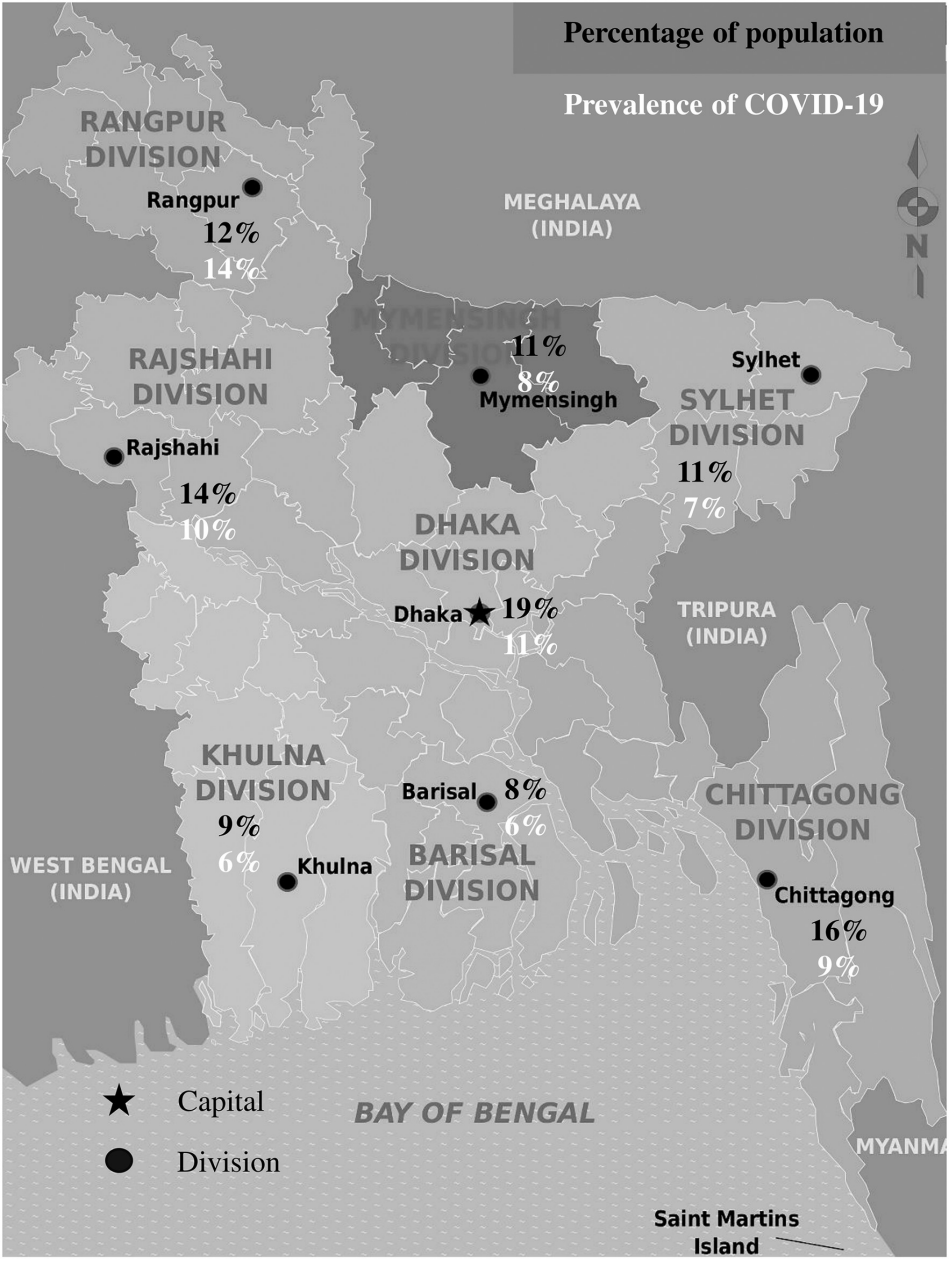
Frequency distribution of the study participants and prevalence of COVID-19 in Bangladesh.

In this study, 1690 participants were enrolled during the second wave of COVID-19 in Bangladesh. Participants were enrolled randomly during the study period from 54 districts. Male to female ratio of the participants was 1.2:1. The mean age of the study population was 34±3.9 years. The study population was distributed into seven age groups (Table 1). The most prevalent age group was 20–29 years (41.9%). The highest frequency of the participants were from Dhaka (19%) followed by Chittagong (16%) (Fig 1). Among 1690 participants, 202 (11.95%) were tested positive for COVID-19. Prevalence of COVID-19 was highest in Rangpur (14%), followed by Dhaka (11%) (Fig 1).

**Table 1:** Sociodemographic characteristics of the study population.

Variables	Male	Female	Total
Study Population	54.7% (924 of 1690)	45.3% (766 of 1690)	100% (1690 of 1690)
**Age (in years)**			
Below 10	66.7% (8 of 12)	33.3% (4 of 12)	.7% (12 of 1690)
10–19	58% (178 of 307)	42% (129 of 307)	18.2% (307 of 1690)
20–29	56.7% (401 of 708)	43.3% (307 of 708)	41.9% (708 of 1690)
30–39	57.5% (162 of 282)	42.5% (120 of 282)	16.7% (282 of 1690)
40–49	57.6% (151 of 262)	42.4% (111 of 262)	15.5% (262 of 1690)
50–59	64.7% (55 of 85)	35.3% (30 of 85)	5% (85 of 1690)
Above 60	61.8% (21 of 34)	38.2% (13 of 34)	2% (34 of 1690)
**Monthly Income (Thousands in taka)**			
Less than 10	59.6% (372 of 624)	40.1% (252 of 624)	36.9% (624 of 1690)
10–29	54.2% (156 of 288)	45.8% (132 of 288)	17% (288 of 1690)
30–49	62.6% (67 of 107)	37.4% (40 of 107)	6.3% (107 of 1690)
50–79	56.2% (18 of 32)	43.8% (14 of 32)	1.9% (32 of 1690)
More than 80	53.8% (21 of 39)	46.2% (18 of 39)	2.4% (39 of 1690)
No information	53.7% (322 of 600)	46.3% (278 of 600)	35.5% (600 of 1690)
**Occupation**			
Physician	54% (93 of 172)	44% (79 of 172)	10.2% (172 of 1690)
Teacher	59.6% (53 of 89)	40.4% (36 of 89)	5.3% (89 of 1690)
Researcher	57.1% (4 of 7)	42.9% (3 of 7)	0.4% (7 of 1690)
Farmer	62.5% (217 of 347)	36.5% (130 of 347)	20.5% (347 of 1690)
Student	54.9% (456 of 831)	44.1% (375 of 831)	49.2% (831 of 1690)
Police	58.3% (35 of 60)	41.7% (25 of 60)	3.5% (60 of 1690)
Businessman	56.9% (41 of 72)	43.1% (31 of 72)	4.3% (72 of 1690)
Others	60.7% (68 of 112)	29.3% (54 of 112)	6.6% (112 of 1690)

### The tendency of the participants to follow the preventive measures

The association between demographic factors and the likelihood of following the preventive health measures was determined by the Chi-square statistics. Age, gender, occupation and monthly income of the participants influenced the behaviors of practicing the preventive health measures significantly (Table 2). The likelihood of using face masks was significantly associated with the male (*p* = .001) participants aged >60 years (*p* = .001), monthly income of 50–79 thousand taka and with profession of physician (*p* = .002). Practice of hand sanitization and cleaning hands were also significantly associated with gender (male, *p* = .005; female, *p* = .041), age (20–29 years, *p* = .05; 30–39 years, *p* = .03; 40–49 years, *p* = .04; >60 years *p* = .05), monthly income (50–79 thousand taka, *p* = .05) and occupation (physician, *p* = .01) (Table 2). The practices of maintaining social distances, avoiding crowded places and following lockdown were also related with age, gender, occupation and monthly income of the participants (Table 2).

**Table 2:** Association between the demographic factors and the likelihood of following the preventive measures.

Variables		Preventive measures (*p* value)				
		Face mask using	Hand sanitization	Maintaining social distances	Avoiding crowded places	Following lockdown
**Gender**	Male	.001	.005	.05	.028	.017
	Female	.024	.041	.004	.037	.05
**Age (in years)**	Below 10	.074	.84	.56	.64	.89
	10–19	.48	.45	.43	.04	.49
	20–29	.021	.05	.04	.034	.53
	30–39	.004	.03	.03	.001	.024
	40–49	.024	.04	.001	.05	.04
	50–59	.04	.001	.05	.04	.06
	Above 60	.001	.05	.03	.001	.05
**Monthly income (Thousands in taka)**	Less than 10	.07	.84	.86	.054	.45
	10–29	.04	.37	.63	.04	.61
	30–49	.05	.45	.05	.05	.34
	50–79	.005	.05	.004	.001	.05
	More than 80	.01	.03	.034	.034	.38
	No information	.08	.75	.46	.82	.68
**Occupation**	Physician	.002	.01	.003	.001	.03
	Teacher	.021	.04	.04	.04	.14
	Researcher	.05	.019	.01	.34	.02
	Farmer	.08	.64	.37	.07	.86
	Student	.074	.035	.42	.64	.34
	Police	.065	.74	.84	.42	.75
	Businessman	.03	.37	.67	.16	.61
	Others	.049	.57	.49	.21	.47

Chi-square test was performed among the variables (*N* = 1690). *P* value < .05 was considered statistically significant.

### Practice of health behaviors of the participants amid the pandemic

**Fig 2:**
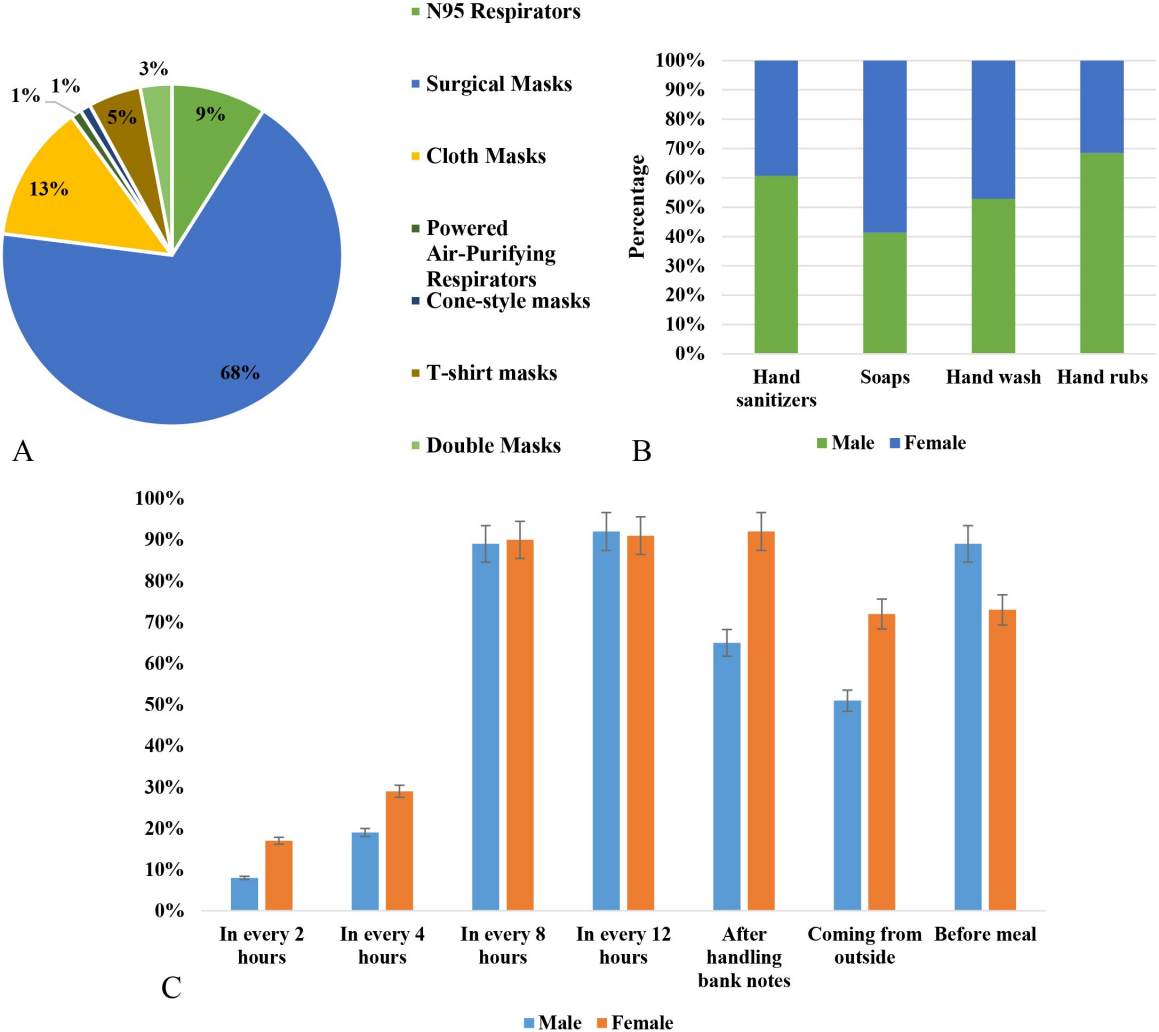
A. Trends of the participants to use different types of face masks, B. Gender distribution of the practices of using different types of hand sanitizing media, C. Frequency distribution of the practices of hand cleaning among male and female in Bangladesh.

**Fig 3:**
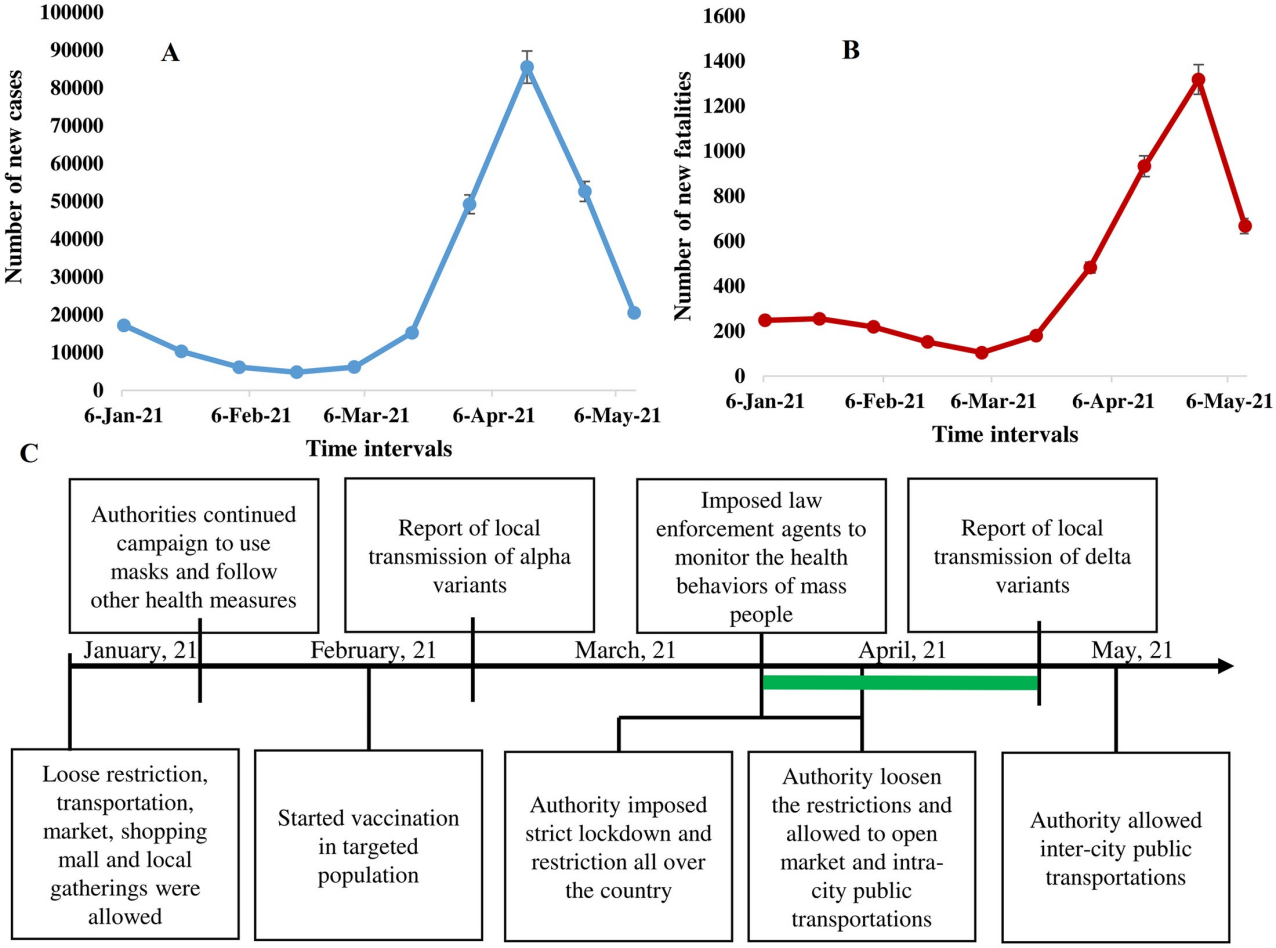
Distribution of COVID-19 A. Cases and B. Fatalities in Bangladesh during the second wave, C. Chronology of taken measures by the authorities against COVID-19 during the second wave.

**Fig 4:**
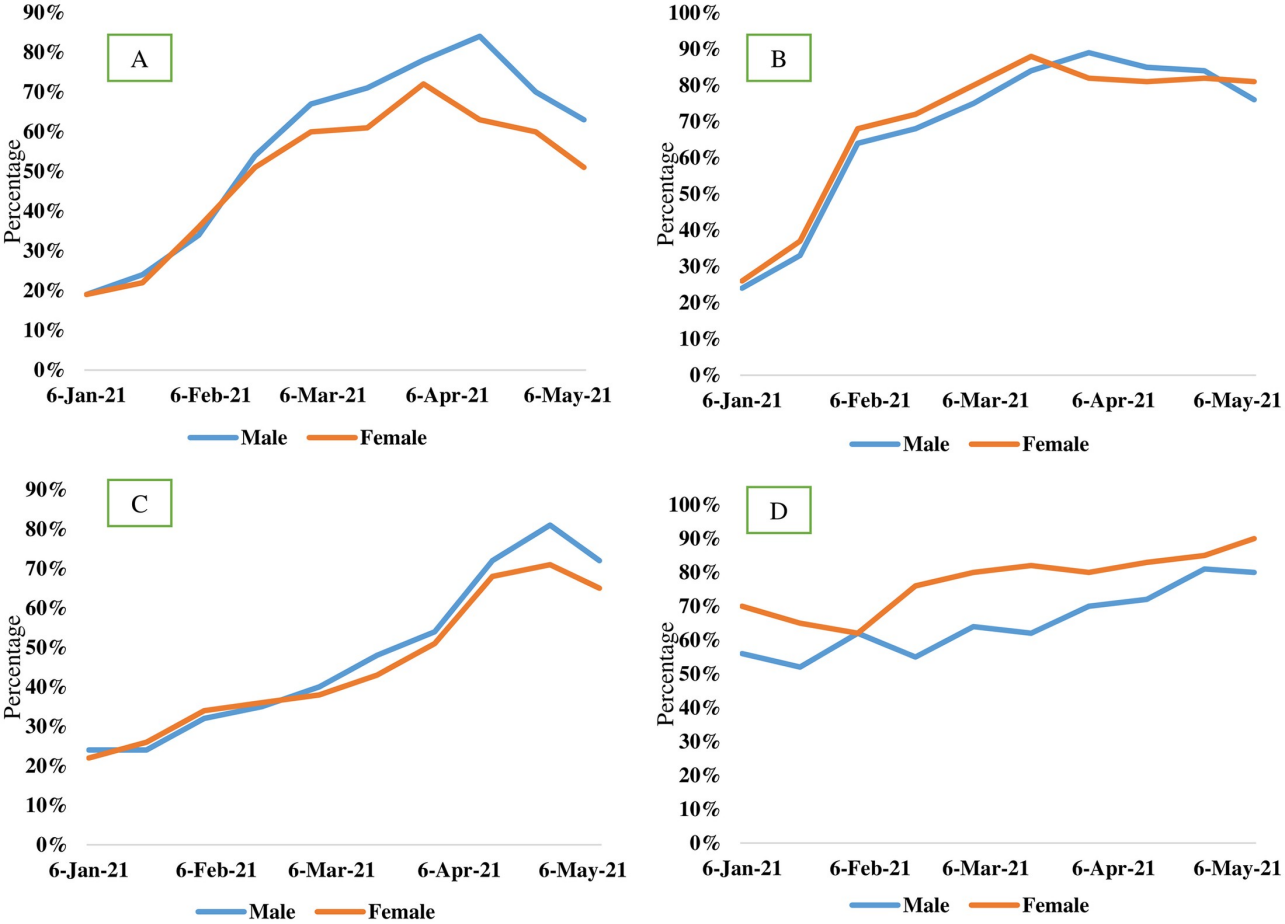
Gender-wise changes of the frequency of A. Using face masks, B. Cleaning hands, C. Maintaining social distance and D. Avoiding hand shaking among the participants with the progress of the second wave.

We analyzed the association of the likelihood of following the preventive measures and the health outcomes of COVID-19 during the second wave in Bangladesh. About 68% (1149 of 1690) participants used one time surgical masks, and 13% (220 of 1690) participants used cloth masks (Fig 2). Further, about 68% male used hand rubs and 60% used hand sanitizers in outside, after returning from outside and after handling bank notes. Among the female participants, about 60% used soaps and 48% used hand wash. Male participants were more conscious in keeping hands clean than the female (Fig 2). Restrictions on national and international travel and transportations were loosen. The continuous progress of cases and fatalities in Bangladesh and actions taken by the authorities are depicted in Fig 3. The authorities imposed strict restrictions related with COVID-19 from the beginning of the second wave of the pandemic. During the second wave, due to the lockdown and law enforcement, the tendency of the people to follow the health guidelines increased by 50%-100%. The frequency of the participants to use masks outside increased from 18% in January to 85% in April, 2021. The frequency of hand washing practices also increased significantly in both male and female participants. However, the tendency to maintain social distance increased during the middle of the second wave (Fig 4). About 70% male and 75% female avoided hand shaking during the study period (Fig 4). Implementation of restrictions and health guidelines during the initial stage of the second wave help in reducing the rapidity and duration of the second wave by 30–55% in Bangladesh (Fig 3).

### Preventive measures and COVID-19 associated health outcomes

The Chi-square test was conducted to determine the relationship between the protective measures and reduction of infection and death of COVID-19. The association between the preventive health measures and health outcomes of COVID-19 was significant (*p* = .001). The participants using masks appropriately had lower probability of getting infection (*p* = .001). Further, the participants using face masks properly were less likely to develop serious health conditions than the participants not using face masks (Table 3). The participants washing and cleaning hands by soaps/hand sanitizers appropriately had reduced risk of infection (*p* = .005), hospitalization (*p* = .02), ICU admission (*p* = .05) and death (*p* = .005). Of note, new cases and fatalities of COVID-19 reduced significantly among the participants maintaining social distance in outside (*p* = .03 and *p* = .01), avoiding gatherings (*p* = .001 and *p* = .005), long distance travels (*p* = .05 and *p* = .04), hand shaking (*p* = .05 and *p* = .01) and following restrictions associated with COVID-19 (*p* = .002 and *p* = .03). Health practitioners including doctors, nurses, technicians and ward boys using PPE and hand gloves during the duty hours had significantly reduced chances of infection (*p* = .02) and death (*p* = .001) due to COVID-19. On the contrary, the participants not following the preventive health measures were more likely to get infected, hospitalized and death due to COVID-19 (Table 3).

**Table 3:** Measures of association among the preventive measures and COVID-19 related health outcomes.

Variables	Reduction of COVID-19 related outcomes (*p* value)				
Practices	Parameter	New cases	New fatalities	Hospitalization	Admission to ICU
**Mask using**	Appropriately	.001	.01	.02	.05
	Moderately	.26	.04	.047	.04
	Not using	.84	.72	.91	.47
**Hand washing/hand sanitizer using**	Appropriately	.005	.005	.02	.05
	Moderately	.034	.03	.04	.02
	Not using	.67	.72	.31	.51
PPE/Hand gloves using*	Appropriately	.02	.001	.006	.05
	Not using	.89	.43	.73	.62
**Social distance maintaining**	Appropriately	.03	.01	.05	.001
	Moderately	.05	.06	.05	.025
	No	.93	.34	.82	.38
**Avoiding hand shaking**	Yes	.05	.01	.05	.04
	No	.42	.67	.52	.39
**Avoiding gatherings**	Yes	.001	.005	.01	.05
	No	.38	.27	.64	.51
**Avoided long distance movement**	Yes	.05	.04	.02	.05
	No	.54	.47	.35	.62
**Following government restrictions**	Yes	.002	.03	.05	.04
	No	.24	.54	.67	.98

Chi-square test was performed among the variables (N = 1690). *P* value < .05 was considered statistically significant.

### Logistic regression analyses

In the regression analysis, both multivariate and univariate analysis were conducted. Among the sociodemographic factors, age, sex, monthly income and access to health facilities were considered for the analysis. Male participants had higher odds of infection (OR: 1.91, 95% CI: 1.13–2.74). Further, the participants aged above 40 years had higher odds of infection (OR: 2.93, 95% CI: 1.95–4.24) of COVID-19. However, participants with better access to health facilities and guidelines had lower odds of infection (OR: 0.71 95% CI: 0.39–1.61). The participants following the health guidelines appropriately had lower risks of infection and death. Participants using masks properly had a lower odds of infection (OR: 0.02, 95% CI: 0.01–0.43) during the second wave. The risk of getting infected was also low in people maintaining social distances (OR: 0.04, 95% CI: 0.01–0.33), avoiding crowded places (OR: 0.07, 95% CI: 0.02–0.19) and avoiding hand shaking (OR: 0.17, 95% CI: 0.09–0.41) (Table 4). Doctors and health workers using PPE and hand gloves in duty hours had reduced risks of infection (OR: 0.10, 95% CI: 0.04–0.63). In multivariate regression model, this study also found that preventive health measures had reduced the risks of infection among the study population. In age adjusted odds ratio, lower risk of infection was detected in mask users (aOR: 0.04, 95% CI: 0.02–0.43), people keeping hands clean (aOR: 0.46, 95% CI: 0.27–0.97) and maintaining social distances (aOR: 0.23, 95% CI: 0.17–0.91) (Table 4).

**Table 4:** Logistic regression analyses among the preventive measures and different parameters of COVID-19 pandemic.

**Univariate analysis**		
**Variables**	**OR (95% CI)**	***p* value**
Gender	1.91 (1.13–2.74)	.005
Age	2.93 (1.95–4.24)	.002
Better access to health facilities	0.71 (0.39–1.61)	.486
High income	1.23 (0.46–2.67)	.001
**Health practices**		
Wearing masks outside	0.02 (0.01–0.43)	.041
Using hand sanitizers/soap/hand wash	0.18 (0.09–0.93)	.013
Maintaining social distance outside	0.04 (0.01–0.33)	.005
Reduced outside activity	0.07 (0.02–0.19)	.001
Reduced hand shaking	0.17 (0.09–0.41)	.017
Using PPE and hand gloves (health professionals)	0.10 (0.04–0.63)	.341
Avoiding inter-city movement	0.39 (0.75–2.30)	.001
Following government guidelines for COVID-19 pandemic	0.41 (0.12–0.89)	.049
**Multivariate analysis**		
**Variables**	**Adjusted OR (95% CI)**	***p* value**
Age: >40 years vs. <40 years	2.47 (1.14–4.74)	.004
Residence: Urban areas vs. village areas	2.74 (1.43–5.06)	.037
Better access to health facilities vs. worse access to health facilities	0.43 (0.12–0.98)	.647
High income vs. low income	1.76 (0.91–3.87)	.001
Mask users vs non-users	0.04 (0.02–0.43)	.005
Keeping social distance vs no social distance	0.23 (0.17–0.91)	.001
Hand cleaning vs no cleaning	0.46 (0.27–0.97)	.049

*P* value < .05 was considered statistically significant. OR- odds ratio, CI- Confidence intervals

## Discussion

In this study, we analyzed the association of protective health measures in reducing the transmission and severity of COVID-19 during the second wave. During the study period, the alpha, beta and delta variants of SARS-CoV-2 were circulating in Bangladesh. This study found that implication of strict measures against COVID-19 increased the likelihood of the participants (about 50%-100%) to follow the rules of using masks outside, maintaining social distances, avoiding hand shaking and sanitizing hands. Sociodemographic factors including age, gender, monthly income and occupation were significantly associated with the likelihood of the participants to follow the preventive health measures amid the pandemic, which are in good agreement with the previous findings [11–13, 24–26]. Significant association was detected between the preventive health measures and reduction of health outcomes including infection, admission to hospitals, ICU and death associated with COVID-19, (*p* = .001) in Bangladesh. The participants following protective health measures regularly and properly were less likely to get infected and develop serious health conditions by COVID-19. In spite of the circulation of alpha and delta variants, protective health measures provided significant protection against the transmission of COVID-19 in Bangladesh. In this study, we analyzed the association between the protective health measures and reduction of transmission of the disease and the findings are in good agreement with the previous preliminary studies reporting the association of preventive health measures with the severity of the pandemic [11–13, 24–26]. This study reported lower risks of infection by SARS-CoV-2 among the participants using masks properly (OR: 0.04, 95% CI: 0.02–0.43), maintaining social distance (OR: 0.23, 95% CI: 0.17–0.91) and keeping hands clean by using soaps/sanitizers (OR: 0.46, 95% CI: 0.27–0.97). These findings were in similarity with the previous studies [26–31].

The epidemiologic and sociodemographic characterizations of the participants were also conducted. The prevalence of COVID-19 was 11.5% (195 of 1690) among the study population. Male (54.7%, 924 of 1690) was the predominant sex group among the study population. The mean age of the participants was 34±3.9 years. These epidemiologic findings were in good agreement with previous studies in Bangladesh, India and the USA [32, 33]. The sociodemographic factors were involved in determining the transmission and fatality among the study population by affecting the tendency to follow the health rules. The risks of infection increased among male participants aged above 40 years, living in urban areas. These findings are consistent with the previous works demonstrating about the association of sociodemographic factors with the COVID-19 pandemic [32, 33].

Both the previous studies and real life experiences suggest that preventive measures such as using masks properly, avoiding crowded places, crowded indoor settings, maintaining social distance outside and cleaning hands with soap or sanitizers properly and frequently can reduce the risk of transmission. In consistent with these findings, our study suggested that following preventive health measures appropriately can reduce the rate of transmission significantly [11–13, 28–31]. According to the findings of WHO, COVID-19 is transmitted from the infected individuals to the healthy susceptible via small droplets produced during coughing, sneezing, speaking, singing or breathing [34–36]. The preventive health measures probably interrupted the mode of transmission of COVID-19 and reduced the increase of cases and fatalities despite the circulation of alpha or delta variants in Bangladesh.

Emergence of new variants such as alpha, beta, gamma, delta, epsilon, kappa and eta with an altered transmission capability requires more concern over the mode of transmission of SARS-CoV-2. Why some variants are more transmissible than the others is still unknown. Previous studies suggested that the host factors, population density and environmental factors are involved in inducing the emergence of new variants [37, 38]. However, our study suggested that maintaining proper health guidelines and safety measures can significantly reduce the prevalence of COVID-19 caused by any of the alpha, beta or delta variants. These findings are in good agreement with the previous studies [11–13, 28–31]. People’s attitudes toward following the health rules differed greatly with the increase of cases and fatalities. Guidelines provided by the authorities and policy makers were followed by the participants after significant increase in number of cases and fatalities. Further, vaccine against COVID-19 was available to the targeted people for a limited time. Only 0.1% of the participants received vaccine during the study. As a result, the association of vaccines in containing the pandemic in Bangladesh was excluded from this study. This study reported that the transmission rate and cases were reduced significantly when about 70% of the participants used masks properly, washed hands regularly and avoided crowded places. Previous studies have also reported that masks are effective preventive measures to reduce the spread of other infectious virus like influenza virus [39]. These findings were consistent with the national COVID-19 report in Bangladesh [40].

This study was conducted on a relatively large sample to interpret the association of preventive health measures on the reduction of the transmission rate of the COVID-19 pandemic. Another strength of this study was the integrated analyses of data from both the participants and the real life situations. However, there are several limitations to this study. At first, the source tracking of transmission was absent in this study which could identify clearly the sources. Further, we couldn’t determine the prevalence of specific variants among the study participants. This study included self-reported data from the participants which is another limitation. In future, studies with large sample and characterization of the sources of transmission and variants of SARS-CoV-2 should be conducted to create a clear concept on the association of preventive measures in containing the pandemic. Our findings suggest that any variant of COVID-19 can be prevented from spreading by using face masks, hand washing, maintaining social distance, and avoiding crowded places. This study will serve as a comprehensive baseline for future studies aimed at identifying and evaluating appropriate preventive measures to curb COVID-19 cases and fatalities. This study will provide outcomes to the national and international health organizations, as well as policymakers, to help prevent the spread of COVID-19.

## Conclusion

This study found that the health burden and fatalities associated with COVID-19 has increased dramatically in Bangladesh during and after the second wave from April 2021. Appropriate preventive health measures were associated with lower risks of infection and fatalities of COVID-19. Among the preventive health measures, washing/cleaning hands by soaps or hand sanitizers, wearing masks properly, avoiding crowded places and maintaining social distance in public places were significantly associated with the reduced number of cases and fatalities. The findings in this study will provide valuable information for both the national and international policy makers to contain the COVID-19 pandemic.
